# Music Interventions for Early-Stage Cognitive Decline: A Systematic Review and Meta-Analysis

**DOI:** 10.1093/geroni/igab046.2448

**Published:** 2021-12-17

**Authors:** Jennie Dorris, Stephen Neely, Juleen Rodakowski

**Affiliations:** 1 University of Pittsburgh, Pittsburgh, Pennsylvania, United States; 2 Carnegie Mellon University, Pittsburgh, Pennsylvania, United States

## Abstract

Older adults in the early stages of Alzheimer’s and dementia deserve effective modalities that support their cognition, emotional well-being, and social engagement. Music has demonstrated potential to support these critical outcomes through its ability to simultaneously stimulate multiple areas of the brain and induce neuroplasticity. We reviewed randomized controlled trials for studies that specifically utilized active music-making interventions for older adults with early-stage cognitive decline to assess their effects on cognition, emotional well-being, and social engagement. Additionally, this review categorized the specific music activities employed by each intervention. We conducted searches on Medline (Ovid), APA PsycInfo (Ovid) CINAHL (Ebsco), and Embase (Elsevier). Our search yielded 285 potential studies. We analyzed 19 studies with 1,387 participants for potential effect sizes and intervention ingredients. Of the 19 studies, eight studies, recruiting a total of 460 participants, were used to conduct a random-effects meta-analysis to assess the effect of music on cognition. Meta-analytic aggregration of effect sizes showed that music had a modest positive effect on cognition compare with the control conditions (SMD= 0.26; P= 0.008; 95% confidence interval, 0.07, 0.45; I2= 5%). The musical activities of 1) singing and/or playing pre-composed songs and/or 2) creating music in the moment were utilized in the protocols. This research demonstrates that active music-making supports cognition for older adults with early-stage cognitive decline. Future music programs should consider inclusion of pre-composed songs, as well as music creation, to better understand the power for music to provide critical support for a rapidly growing segment of the population.

